# Peripheral Nerve Schwannoma with an Unusual Anatomical Site

**DOI:** 10.3390/reports9040332

**Published:** 2026-10-01

**Authors:** Alina Badlaeva, Natalia Mezhelumova, Natalia Arzhanukhina, Dmitry Bulanov

**Affiliations:** 1 National Medical Research Center for Obstetrics, Gynecology and Perinatology Named After Academician V.I. Kulakov of the Ministry of Health of Russia, Moscow 117513, Russia; 2 Department of Pathology and Clinical Pathology, Pirogov Russian National Research Medical University, Moscow 117997, Russia; 3 Diagnostic Clinical Center №2, Petrovsky National Research Center of Surgery, Moscow 117593, Russia; 4 N.N. Blokhin National Medical Research Center of Oncology, Moscow 115522, Russia

**Keywords:** schwannoma, MRI, vulvar lesion, perianal lesion

## Abstract

We present the case of a 40-year-old woman diagnosed with schwannoma at an infrequent localization. The patient complained of multiple painful lesions in the vulvar region and perianal fold, accompanied by intense, constant pain that slightly increased during menstruation. The initial diagnosis was extragenital endometriosis. Histopathological analysis demonstrated a characteristic proliferation of bland spindle cells arranged in short fascicles containing Verocay bodies, as well as a distinctive immunophenotype, supporting the diagnosis of schwannoma. This case underscores the diagnostic difficulty of distinguishing schwannoma from other mesenchymal tumors and non-tumor lesions at this site using imaging alone and emphasizes the need for histopathological confirmation to reach a definitive diagnosis.

Schwannoma is a well-circumscribed, noninvasive mesenchymal tumor with an indolent course and rare recurrence, which arises from the myelin sheath of peripheral nerves and expresses neurogenic markers [1]. It can occur sporadically or in association with hereditary tumor predisposition syndromes, including NF2-related schwannomatosis, SMARCB1- or LZTR1-related schwannomatosis, and Carney complex, often presenting with multiple nodules at various anatomical sites [2]. Schwannoma may be localized in the head, neck, extremities, posterior mediastinum, and retroperitoneum [3]. However, schwannomas occurring without schwannomatosis account for only 5% of benign tumors identified in the perianal region and are extremely rare in the vulvar region [4,5,6,7].

A 40-year-old woman complained of multiple painful lesions in the left labium major and perianal fold, which had been present for 2 years. They were accompanied by constant pain, slightly worsening during menstruation, that was resistant to nonsteroidal anti-inflammatory drugs. Her surgical history included removal of lesions in the pubic area in 2007 and 2017, for which operative and histological data were unavailable. The patient reported a positive family history of schwannoma—a sibling had a tumor of the upper extremity. Nevertheless, the initial diagnosis was interpreted as extragenital endometriosis based on the vulvar and perianal location; the character of the pain, which was intense, constant, and notably exacerbated during menstruation with radiation to the left thigh, mimicking the cyclical pain pattern of endometriotic implants; and the recurrent clinical course, which was interpreted as recurrent endometriosis after incomplete excision. However, because prior histopathological data were unavailable, true recurrence could not be confirmed.

**Figure 1:**
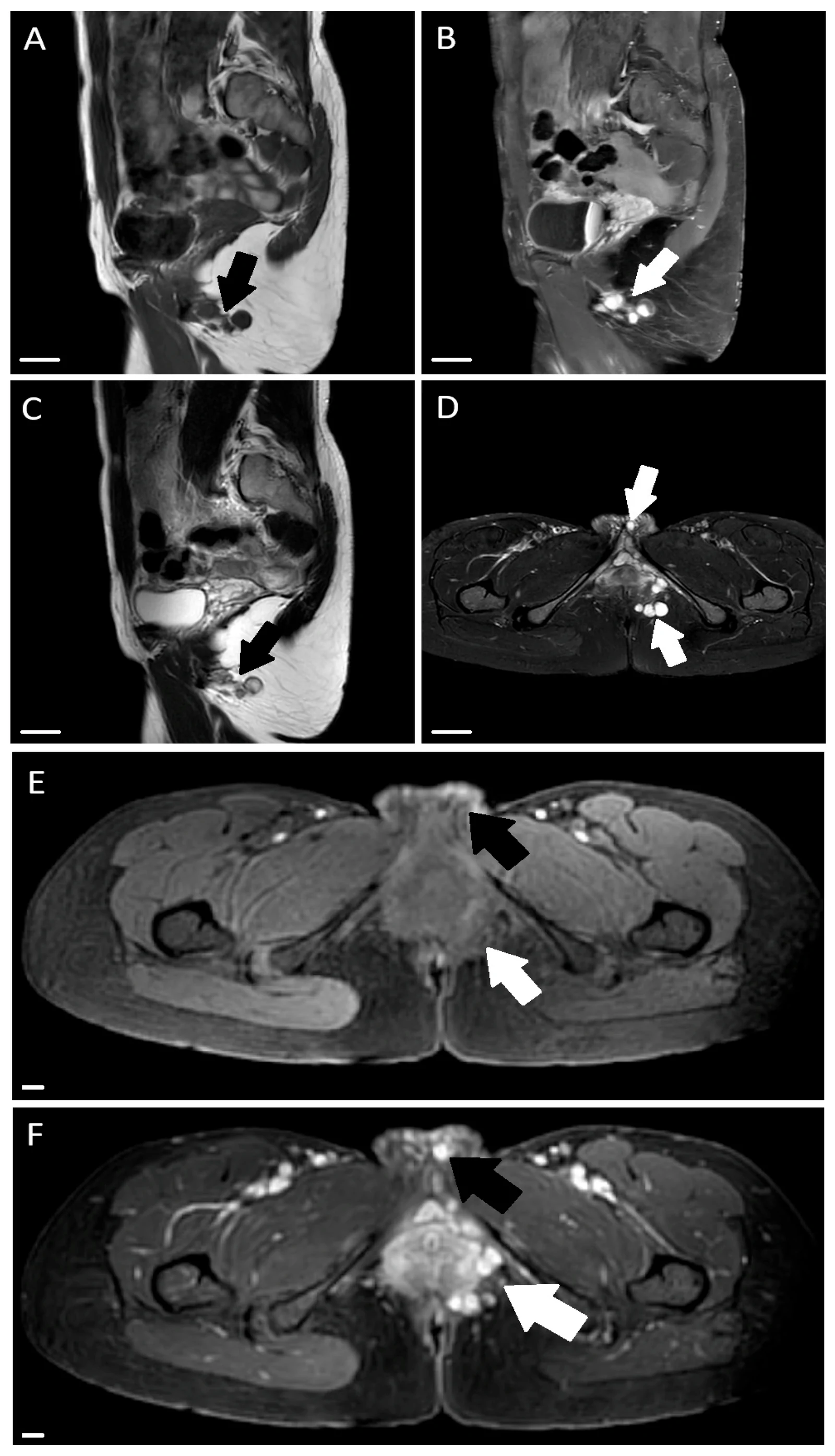
A magnetic resonance imaging (MRI) scan showed multinodular lesions in the soft tissues of the left labium major and left perineum, measuring approximately 1.0 × 1.0 × 1.5 to 4.0 × 3.5 × 2.5 cm: (**A**–**F**) arrows; scale bar 5 cm. The lesions had well-defined margins, with isointense to slightly hypointense signal on T1-weighted images (WI) ((**A**) sagittal plane); hyperintense signal on T2WI with non-contrast fat saturation ((**B**) sagittal plane); hyperintense signal on T2WI with areas of hypointensity ((**C**) sagittal plane); and hyperintense signal on T2WI with non-contrast fat saturation ((**D**) axial plane). The tumors also showed isointense signal on pre-contrast T1WI and prominent homogeneous enhancement after gadolinium administration ((**E**,**F**) axial plane). The patient was then treated with surgical excision through separate local incisions over the vulvar and perianal lesions. No major nerve trunk was encountered, and the lesions were not adherent to named nerves. Gross total excision of all nodules was achieved.

**Figure 2:**
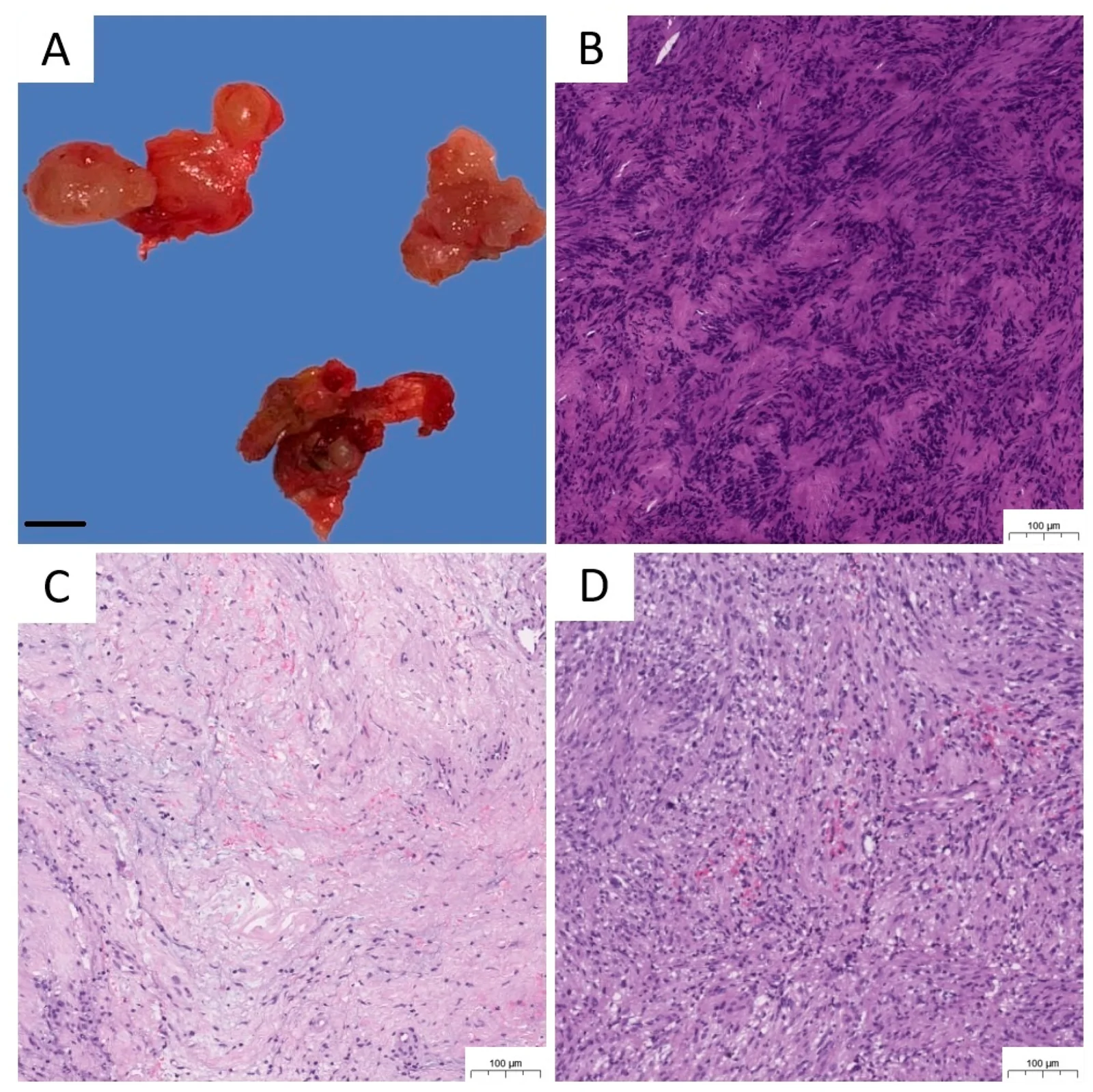
Gross examination showed encapsulated, light-tan, glistening, discrete, well-circumscribed nodules (**A**); scale bar = 1.0 cm. At low-power view, all excised nodules had identical microscopic features. Each nodule had a fibrous capsule and distinctive borders, exhibiting discrete, abrupt interfaces between a cellular Antoni A zone with palisaded Schwann cell nuclei separated by fibrillary eosinophilic material—pathognomonic Verocay bodies (**B**) (100×)—and a myxoid Antoni B zone (**C**) (100×). There was no plexiform growth pattern: the nodules were not interconnected by nerve fascicles, and no network-like arrangement of Schwann cell fascicles was seen. The findings are therefore consistent with multiple separate conventional schwannomas rather than a plexiform schwannoma. A chronic inflammatory infiltrate was also found (**D**) (100×). The surgical margin was negative for all lesions.

**Figure 3:**
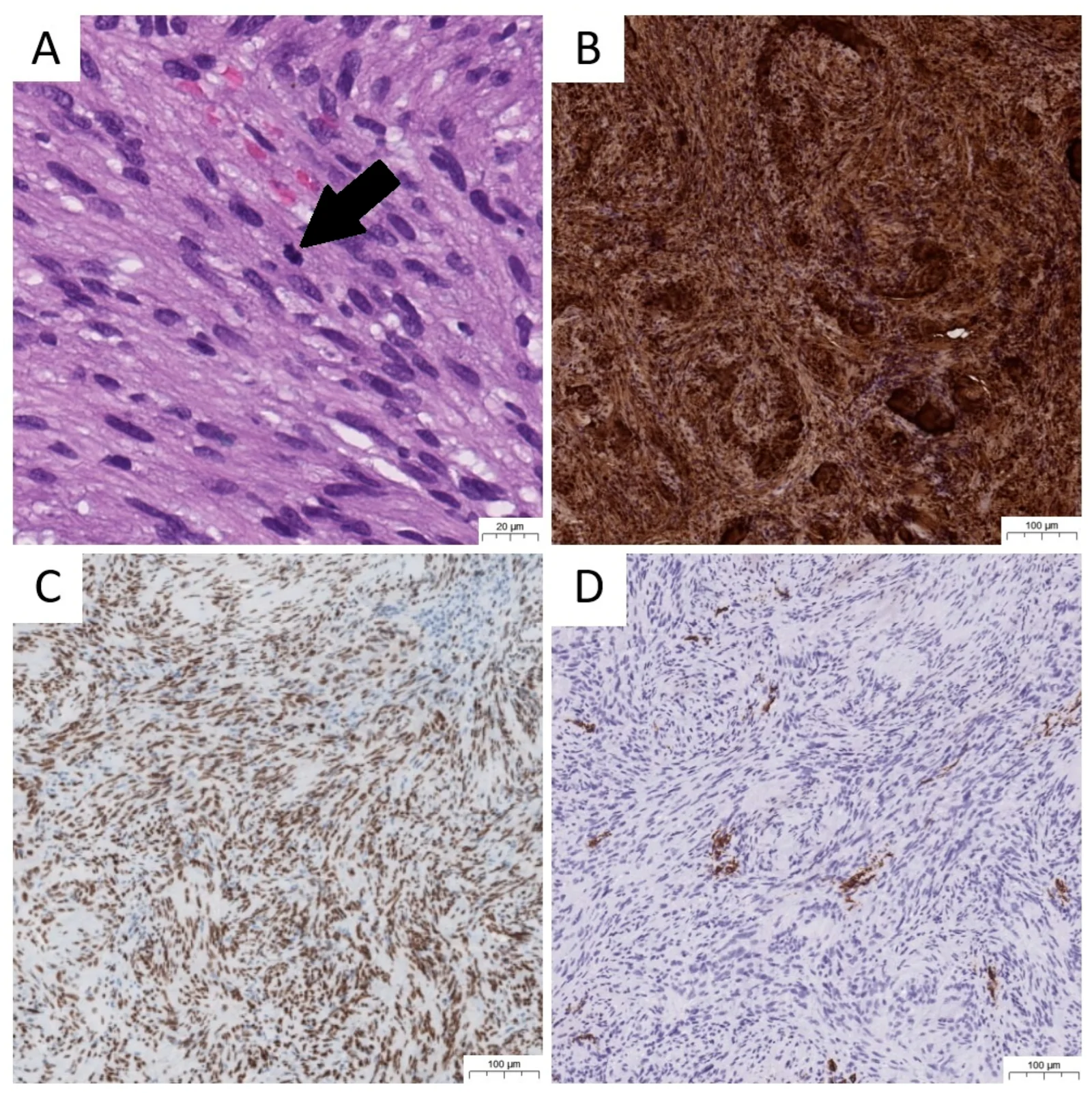
The neoplasm was composed predominantly of spindle cells with elongated, wavy nuclei and indistinct cytoplasmic borders (**A**) (300×). The mitotic rate was 1 mitotic figure per 10 HPF (**A**) (arrow). Immunohistochemistry was performed with the following results. Tumor cells showed strong, diffuse immunoreactivity for S100 (**B**) (200×), as well as strong nuclear staining for SOX10 (**C**) (100×). Tumor cells showed no immunostaining for CD34 (**D**) (100×) or smooth muscle markers. The differential diagnosis included leiomyoma, neurofibroma, cellular angiofibroma, and malignant peripheral nerve sheath tumor (MPNST). The presence of bland spindle cells arranged in short fascicles containing Verocay bodies, along with a distinctive immunophenotype, supported a diagnosis of schwannoma. In contrast, neurofibroma is unencapsulated, hypocellular, lacks a biphasic pattern, and shows only scattered S100-positive cells, reflecting a smaller proportion of Schwann cells. The absence of CD34, desmin, and SMA expression made it possible to exclude cellular angiofibroma and leiomyoma, respectively. MPNST was also considered in the differential diagnosis; however, the absence of significant cytologic atypia, the low mitotic rate, and strong, diffuse S100 and SOX10 positivity supported schwannoma rather than MPNST.

The postoperative recovery was uneventful, and the patient was discharged on day 5 postoperatively. At the 6-month follow-up, the woman underwent an MRI of the chest, abdomen, and pelvis that did not reveal local recurrence or other tumors. She remained completely asymptomatic, with no clinical signs or radiological evidence of local recurrence. No iatrogenic neurological deficits, urinary or fecal incontinence, or perineal sensory disturbances were observed.

Several limitations of this case should be acknowledged. First, although the patient reported a family history of schwannoma, comprehensive genetic testing for schwannomatosis-related genes was not performed, so NF2-related, SMARCB1-related, or LZTR1-related schwannomatosis cannot be excluded. In addition, histopathological reports of the lesions removed in 2007 and 2017 were not available. These factors limit our ability to determine whether the current lesion represents a true recurrence or a de novo tumor, and they preclude a precise estimation of her individual risk of recurrence. Another limitation is that a formal preoperative neurological examination and electrophysiological testing (EMG, pudendal nerve studies) were not performed, as the initial diagnosis was extragenital endometriosis. Brain and spinal MRI were also not performed. A further limitation of this case is the short follow-up period since the last operation, which confirms the absence of early local recurrence but cannot exclude late recurrence or new schwannomas. Therefore, a longer follow-up period is necessary to draw definitive conclusions about recurrence risk and prognosis.

This case illustrates several key challenges in diagnosing and treating schwannomas in this location. First, the clinical presentation was unusual, with constant pain, whereas schwannomas are typically painless [8]. However, large schwannomas and tumors located near major nerve trunks can be symptomatic, presenting with pain, dysesthesia, paresthesia, motor weakness, or focal neurological deficits [3]. In our patient, the intense, constant catamenial pain initially mimicked endometriosis, illustrating this diagnostic pitfall. Similar diagnostic pitfalls have been reported for benign peripheral nerve sheath tumors, with initial misdiagnosis in up to 44.7% of cases [9].

Although the initial clinical diagnosis was extragenital endometriosis, the differential diagnosis of vulvar and perianal soft-tissue masses also included leiomyoma, neurofibroma, lipoma, and Bartholin gland cyst/abscess. On MRI, endometriosis typically appears as T1-hyperintense lesions due to hemorrhagic content, with T2-hypointense or heterogeneous signal and possible contrast enhancement. Leiomyoma is usually T1-isointense, T2-hypointense, and shows homogeneous enhancement. Neurofibroma is T2-hyperintense and may show a target sign and contrast enhancement. Schwannoma is typically T1-iso- to hypointense and T2-hyperintense, with heterogeneous enhancement and possible “target sign,” “split-fat sign,” or “fascicular sign” [10].

Given the patient’s family history of schwannoma and the possibility of an underlying schwannomatosis or NF2, we propose the following management strategy. First, baseline brain and spinal MRI should be performed to rule out other schwannomas, particularly acoustic neuromas and spinal lesions. Second, genetic testing for *NF2*, *SMARCB1*, and *LZTR1* was offered but not performed because the patient declined. Third, clinical follow-up every 6–12 months is recommended, with pelvic MRI at least annually for the first 5 years, given the risk of local recurrence or new lesions. If the patient remains asymptomatic and imaging is stable, the interval may be extended to every 2 years thereafter. Any new neurological symptoms should prompt immediate imaging. Although the optimal surveillance interval for schwannomatosis is not firmly established, we believe a cautious approach is justified in this case.

The preferred diagnostic and therapeutic strategy for schwannoma of the perianal region or vulvar regions is complete excision of the mass rather than needle biopsy due to insufficient samples as well as cellular polymorphism observed in degenerative areas [11]. The latter can be misinterpreted as a malignant tumor, resulting in surgical overtreatment. Therefore, the optimal method is total removal of the tumor with mandatory subsequent histological and IHC examination. However, the proximity of the schwannoma to the vascular plexuses, nerve endings of the vagina, and the anal sphincter requires particular surgical caution and experience to prevent iatrogenic injuries that could lead to bleeding, urinary and fecal incontinence, and loss of sensation in the genital area.

## Data Availability

Original data supporting the reported results are available upon request from the authors.
